# Genome-Editing Strategies for Treating Human Retinal Degenerations

**DOI:** 10.1089/hum.2020.231

**Published:** 2021-03-17

**Authors:** Joel Quinn, Ayesha Musa, Ariel Kantor, Michelle E. McClements, Jasmina Cehajic-Kapetanovic, Robert E. MacLaren, Kanmin Xue

**Affiliations:** ^1^ Nuffield Laboratory of Ophthalmology, Nuffield Department of Clinical Neurosciences, University of Oxford, Oxford, United Kingdom.; ^2^ Oxford Eye Hospital, Oxford University Hospitals NHS Foundation Trust, Oxford, United Kingdom.

**Keywords:** CRISPR, inherited retinal degenerations, gene editing

## Abstract

Inherited retinal degenerations (IRDs) are a leading cause of blindness. Although gene-supplementation therapies have been developed, they are only available for a small proportion of recessive IRD mutations. In contrast, genome editing using clustered-regularly interspaced short palindromic repeats (CRISPR) CRISPR-associated (Cas) systems could provide alternative therapeutic avenues for treating a wide range of genetic retinal diseases through targeted knockdown or correction of mutant alleles. Progress in this rapidly evolving field has been highlighted by recent Food and Drug Administration clinical trial approval for EDIT-101 (Editas Medicine, Inc., Cambridge, MA), which has demonstrated efficacious genome editing in a mouse model of *CEP290*-associated Leber congenital amaurosis and safety in nonhuman primates. Nonetheless, there remains a significant number of challenges to developing clinically viable retinal genome-editing therapies. In particular, IRD-causing mutations occur in more than 200 known genes, with considerable heterogeneity in mutation type and position within each gene. Additionally, there are remaining safety concerns over long-term expression of Cas9 *in vivo*. This review highlights (i) the technological advances in gene-editing technology, (ii) major safety concerns associated with retinal genome editing, and (iii) potential strategies for overcoming these challenges to develop clinical therapies.

## Introduction

Inherited retinal degenerations (IRDs) comprise a heterogeneous group of disorders associated with mutations in more than 250 genes, and they are characterized by degeneration of photoreceptors and/or the underlying retinal pigment epithelium (RPE), which lead to irreversible sight loss (https://sph.uth.edu/RETNET/).^1^ They affect an estimated 1 out of 2,000 people worldwide and are the leading cause of blindness in the working-age population.^2^

Despite remarkable progress made in the development of therapies for these diseases over the past decade, which have culminated in approved gene therapy for the inherited retinal dystrophy, Leber congenital amaurosis (LCA) associated with biallelic mutations in *RPE65* (Luxturna, Hoffmann-La Roche, Basel, Switzerland),^3^ and implantable retinal prostheses for end-stage retinal degeneration (Argus II; Second Sight Medical Products, Inc., Sylmar, CA; and Retinal Implant AG, Reutlingen, Germany), the majority of IRDs remain untreatable.

The retina provides a unique opportunity for developing genetic therapies due to its distinct immunological tolerance and unique visual and surgical access, which allows noninvasive assessment of treatment safety and efficacy.^4^ Adeno-associated viral (AAV) vectors are the most frequently used vehicles for delivering genetic material into cells due to their high tropism for outer retinal cells and good safety profile.^5^ In addition to Luxturna, AAV-mediated gene supplementation therapies to deliver working copies of disease-associated genes into retinal cells in recessive retinal degenerations, for instance, choroideremia and *RPGR*-associated X-linked retinitis pigmentosa, are being evaluated in advanced-stage clinical trials with promising safety and efficacy data.^6,7^

However, due to a maximum total coding capacity of ∼4.8 kb, many large disease-associated genes cannot be delivered by using a single AAV vector, including *ABCA4* (6.8 kb) in Stargardt disease and *USH2A* (15.6 kb) in Usher syndrome type 2.^8^ In addition, gene supplementation has limited utility in autosomal dominant retinal diseases in which the mutant proteins exert toxic gain-of-function or dominant negative effects.

Antisense oligonucleotides (ASOs) that alter splicing or target mRNA for degradation are currently being explored to treat inherited retinal diseases caused by such mutations.^9^ Genome editing offers another promising alternative approach to address this therapeutic gap by targeted correction of pathogenic mutations within retinal cells. Correcting the genomic mutations is likely to result in more physiological levels of expression, as the gene would be subject to its native transcriptional regulation and epigenetic environment.

The emergence of clustered-regularly interspaced short palindromic repeats (CRISPR)-based gene-editing tools and mature viral vector-mediated delivery methods have made it theoretically possible to correct a wide range of genetic mutations in the retina. Rapid developments in the gene-editing field have already resulted in a Food and Drug Administration-approved human trial of a CRISPR genome-editing therapy for LCA type 10 (LCA10), an inherited retinal dystrophy associated with a common deep-intronic mutation in *CEP290* that causes aberrant splicing and often leads to blindness in early infancy.^10^

Nonetheless, major challenges and questions remain to the development of retinal gene-editing therapies. A viable strategy needs to be suitable for retinal delivery, have limited off-target and immunogenic effects, therapeutic level of on-target editing efficiency, and ideally be adaptable to treating a broad range of genetic mutations. Herein, we review promising therapeutic retinal gene-editing strategies, discuss the main challenges and potential solutions, and propose optimal approaches to cover the heterogeneity of mutations associated with IRDs.

## Classic Crispr-Cas9-Based Genome Editing

Genome engineering using zinc finger nucleases or transcription activator-like effector nucleases have existed for some time, but the clinical application of these techniques has been limited by the generally low *in vivo* editing efficiencies.^11^ The discovery of RNA-guided Cas9 endonuclease from the bacterial CRISPR–CRISPR-associated (Cas) adaptive immune system in 2012 has revolutionized the field of molecular biology and gene therapy.^12^

The CRISPR–Cas system encompasses a variety of components that differ in mechanisms of action and offer therapeutic potential by direct genome interaction and/or editing. Broadly, there are two classes of CRISPR systems, each containing multiple CRISPR types. Class 1 contains type I, type III, and type IV systems; whereas Class 2 contains types II, V, and VI CRISPR systems.^13^ The most widely used platform for genome targeting applications is derived from the Class 2 type II Cas enzyme, Cas9, which acts as a single effector protein; in contrast, the Class I Cas enzymes operate as multi-subunit protein complexes. Despite the complexity of the Cas family, all systems share a requirement for CRISPR RNA (crRNA) for defined target specificity whereas type II variants have an additional requirement for trans-activating RNA, which forms a scaffold structure.

For gene-editing applications, the two crRNAs described earlier are joined as one single guide RNA (sgRNA), which greatly simplifies delivery. The Cas9:sgRNA complex interrogates DNA for the appropriate protospacer adjacent motif (PAM), a short motif adjacent to the target sequence. On recognition of the PAM sequence, the double-stranded DNA unwinds, allowing the Cas9-associated sgRNA to hybridize with the exposed DNA strand (the protospacer). If the DNA sequence matches the sgRNA target sequence, the HNH and RuvC catalytic domains of the endonuclease cleave both strands of the target DNA, generating a double-strand break (DSB).^12^

One constraint of Cas9 is its dependency on the aforementioned PAM sequence to bind target DNA. Because of its simple 5′-NGG-3′ (where N is any nucleotide) PAM sequence requirement, *Streptococcus pyogenes* Cas9 (SpCas9) is used in many different applications. However, researchers are actively exploring other CRISPR systems to identify Cas9-like effector proteins that may have differences in their sizes, PAM requirements, and targeting specificity. Naturally occurring Cas9 variants are large proteins that create limitations when it comes to packaging and delivery into target cells. For example, the SpCas9 coding sequence is 4,098 base-pairs (bp), making therapeutic delivery challenging due to the limited packaging capacity of AAV vectors.^14^ To this end, the discovery of smaller variants such as the 3,246 bp Cas9 from *Staphylococcus aureus* (SaCas9) and the 2,952 bp Cas9 from *Campylobacter jejuni* (CjCas9) provides great therapeutic potential.^5^ In addition to their smaller sizes, both SaCas9 and CjCas9 variants have different PAM requirements, expanding the genome targeting repertoire.

Although the exploration of natural Cas diversity provides one avenue for expanding and improving PAM coverage, the efficiency of Cas activity varies, and to date, no orthologs have demonstrated a marked increase in efficiency compared with the well-characterized SpCas9. Thus, complementary evolution-based and structure-guided engineering approaches have been employed to modify and improve SpCas9 effector scope.^15^

**Figure 1.**
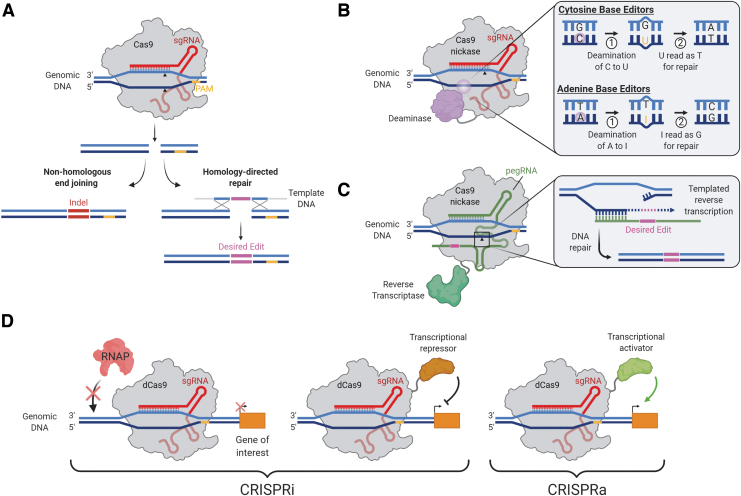
Therapeutic CRISPR-based genome-editing strategies. **(A)** CRISPR-Cas9-induced double-strand breaks. In eukaryotic cells, breaks are repaired through either the error-prone non-homologous end-joining pathway, leading to indel mutations, or the high-fidelity homology-directed repair pathway, which may be leveraged to introduce precise edits in dividing cells. *Black triangles* indicate Cas9 cut sites. **(B)** DNA editing with Base Editors. Base editors consist of a Cas9 nickase fused to a DNA deaminase and use an sgRNA to locate a target sequence, where the deaminase either converts C to T, via U (cytosine base editors) or A to G, via I (adenine base editors). **(C)** DNA editing with Prime Editors. Prime Editors use a Cas9 nickase fused to a reverse transcriptase, with a pegRNA. The nicked DNA strand base pairs with the 3′ end of the pegRNA, which templates the desired DNA edit to be introduced by reverse transcription. **(D)** CRISPRi and CRISPRa. A catalytically dCas9 is guided by a sgRNA to locate a regulatory region of a gene of interest. CRISPRi involves either steric exclusion of RNAP by the dCas9 (*left*) or fusion of a transcriptional repressor (*center*) to reduce gene expression, whereas CRISPRa fuses a transcriptional activator to dCas9 to upregulate gene expression (*right*). CRISPR, clustered-regularly interspaced short palindromic repeats; Cas, CRISPR-associated; CRISPRi, CRISPR-interference; CRISPRa, CRISPR-activation pegRNA, prime editing guide RNA; RNAP, RNA polymerase; dCas9, dead Cas9; sgRNA, single guide RNA. Color images are available online.

In eukaryotic organisms, Cas9-induced DSBs are repaired by either error-prone non-homologous end-joining (NHEJ) or by relatively error-free homology-directed repair (HDR).^11^ Although NHEJ results in random insertions or deletions (indels) and thus gene disruption in the target region, HDR can be harnessed to insert a specific DNA template for precise restoration of the genomic DNA sequence (Fig. 1A). In theory, HDR-mediated repair of the mutant allele using the wild-type allele or another template could correct most mono-allelic mutations in IRDs. However, precise gene modification relying on the HDR pathway has been shown to be ineffective in non-dividing cells such as those present in the retina, and that Cas9-induced DSBs lead to indel mutations at a substantial frequency that annuls the potential benefit from corrective mutations.^11^

Thus, the NHEJ repair pathway is currently the most commonly adopted approach in developing treatments for retinal diseases, especially for mutations arising from autosomal dominant diseases. Through careful design of the guide RNA, the mutant allele can be preferentially disrupted whereas the wild-type allele remains preserved. In the absence of haploinsufficiency mechanisms, this could ameliorate disease phenotypes and has been successfully employed to knock out the mutant rhodopsin (*Rho*) gene in rodent models of autosomal dominant retinitis pigmentosa (adRP) (Table 1).^16,17^ As another example, CRISPR-mediated allele-specific knockdown could be extrapolated to treating the autosomal dominant founder mutation (S163R) in the *C1QTNF5* gene, which is known to be strongly expressed by the RPE and to cause late-onset retinal degeneration.^18^

**Table 1.** *Studies of successful* in vivo *genome editing using CRISPR-Cas systems*

Disease	Target Gene	Mutation	Cas9 Ortholog and Delivery	Editing Mechanism	Animal Model	Main Results	References
adRP	*Rho*	S334ter	SpCas9, plasmid electroporation	Allele-specific knockdown by indel	S334ter-3 rats (carrying mouse *Rho*^S344^)	Nine-fold increase in photoreceptor nuclei 53% Improvement in the optokinetic response	^16^
adRP	*Nrl*	—	SpCas9, two AAV8 vectors	Knockdown by indel (reprogram rods to cone-like cells)	*Rho^−/−^* and *rd10* mice	25% Increase in cone photoreceptor preservation and electroretinogram B waves amplitude by ∼60%	^57^
adRP	*Rho*	P23H	SpCas9-VQR, plasmid electroporation	Allele-specific knockdown by indel	*Rho*^P23H/+^ knock-in mice	Increase in wild-type mRNA by ∼20% compared with untreated control Delayed outer nuclear layer degeneration	^17^
adRP	*Rho*	P23H	SpCas9, two AAV8 vectors	Allele-specific knockdown by indel and wild-type supplementation	*Rho*^P23H/P23H^ and *Rho*^P23H/+^ knock-in mice	Preserved electroretinogram B-waves and outer nuclear layer thickness in Cas9-treated mice compared with mice only given gene supplementation	^58^
arRP	*Mertk*	1.9 kB deletion (intron 1–exon 2)	SpCas9, two AAV8 or 9 vectors	HITI-mediated insertion	RCS rats	Electroretinogram showing improved rod and cone responses compared with untreated and HDR-treated controls	^20^
arRP	*Pde6b*	Y347X	SpCas9/RecA, plasmid electroporation	Induce HDR using sgRNA-targeted RecA	*rd1* mice	Increased survival of rod photoreceptors five-fold compared with nontreated controls	^19^
LCA10	*CEP290*	c.2991 + 1655A > G (IVS26) deep intronic mutation	SaCas9, single AAV5 vector	Inversion/deletion between two sites	IVS26 knock-in mice and NHPs	Editing rate of 27.9 ± 20.7% at 1 × 10^12^ vg/mL vector dose in NHPs Efficacy and tolerance shown in NHPs	^10^
Wet AMD	*Hifa1a*	—	CjCas9, single AAV9 vector	Knockdown by indel	C57BL/6J mice	58 ± 12% efficiency for indel Reduced choroidal neovascularization area by 20 ± 4%	^59^

CRISPR, clustered-regularly interspaced short palindromic repeats; Cas, CRISPR-associated; adRP, autosomal dominant retinitis pigmentosa; arRP, autosomal recessive retinitis pigmentosa; LCA, Leber congenital amaurosis; AMD, age-related macular degeneration; Sa, *Staphylococcus aureus*; Sp, *Streptococcus pyogenes*; Cj, *Campylobacter jejuni*; HITI, homology-independent targeted integration; HDR, homology-directed repair; RecA, recombinase A (from *Escherichia coli*); RCS, Royal College of Surgeons; NHP, non-human primate.

The limited precision and control over NHEJ reduces productive editing efficiency, restricting use to mutations corrected by inversion, deletion, or small indels. In cases of haploinsufficiency, NHEJ could inactivate the mutant allele but wild-type gene supplementation would still be required. Thus, precise gene editing by HDR may be more desirable, allowing greater control over the editing outcome and correction of a greater range of mutations. To overcome the natural lack of the HDR activity in terminally differentiated retinal cells, HDR has been induced in photoreceptors by expressing *Escherichia coli* recombinase A (RecA) with Cas9^19^ (Table 1), though the presence of RecA may increase immunogenicity.

A recently developed approach called homology-independent targeted integration (HITI) may further expand the scope of retinal diseases that can be corrected by CRISPR. Rather than relying on inefficient HDR-mediated repair, HITI expropriates the NHEJ pathway to enable targeted insertion of large genetic fragments in terminally differentiated cells. In the Royal College of Surgeons mouse model of autosomal recessive retinitis pigmentosa caused by a 1.9 kB deletion in the *Mertk* gene, HITI-based CRISPR-Cas9 delivered via dual AAV vectors showed effective editing and improved rod-cone response compared with HDR-treated controls^20^ (Table 1).

Delivery of multiple guide RNAs at separate sites can allow for multiplex editing, and this approach has been utilized to excise deleterious mutations from the genome while maintaining the open reading frame. A recent study showed that a pair of sgRNAs coupled with SpCas9 were highly efficient at excising or inverting a segment of intron 26 that contains a common deep-intronic mutation (c.2991 + 1655A > G) in *CEP290* associated with LCA10, thus restoring normal splicing between exon 26 and exon 27 *in vivo*^10^ (Table 1). The editing efficiency met the targeted therapeutic threshold of 10% and was translatable to non-human primates (NHPs).

Importantly, this study demonstrated several salient features. Peak sgRNA expression was maintained for the duration of the study (40 weeks); Cas9 expression driven by the human rhodopsin kinase (*GRK1*) promoter was photoreceptor specific; therapeutic dose alignment between mice and NHPs provided a dosing strategy for clinical trials; and there was limited drug-induced immunogenicity. Collectively, these findings suggest promoter productivity and Cas9 tolerance, providing a basis for moving other genome-editing therapeutics toward the clinic. Based on these preclinical results, Editas Medicine, Inc. (Cambridge, MA) initiated a phase 1/2 clinical trial of EDIT-101 for the treatment of LCA10 (ClinicalTrials.gov ID: NCT03872479), marking the first clinical application of CRISPR-mediated gene editing in the retina.

## Strategies for Extending the Editing Capability of Crispr-Cas

### DNA base editing and prime editing

Although HDR-mediated gene editing can be harnessed to insert a specific DNA template for precise restoration of the DNA sequence, this pathway is characterized by low efficiency and high rates of undesired indel mutations that nullify the potential benefit from repairing the mutation. Recently, CRISPR-Cas-mediated single base pair editing systems (or “base editing” systems) have been devised to bypass these limitations. Two classes of DNA base editors have been described: cytosine base editors and adenine base editors^21^ (Fig. 1B). Base editors encompass two key components: a Cas9 nickase (nCas9) with one inactive nuclease domain to provide programmable DNA target binding and induce a single-stranded DNA nick, and a single-stranded DNA-modifying enzyme (either cytosine or adenine deaminase) for targeted nucleotide alteration. Collectively, all four transition mutations (C → T, T → C, A → G, and G → A) can be introduced with the available CRISPR-Cas base editors. One of the limiting factors for the application of base editing is the presence of multiple “bystander” cytidines or adenines in the target region, which may also be deaminated. In addition, the target C or A needs to be located within about 15 nucleotides (nt) of a PAM sequence.

Prime editors are the latest addition to the CRISPR genome-engineering toolkit and represent a novel approach to expand the scope of donor-free precise DNA editing to not only all transition and transversion mutations but also small indels.^22^ Prime editors use an engineered reverse transcriptase fused to Cas9 nickase and a prime editing guide RNA (pegRNA). Importantly, the pegRNA differs significantly from regular sgRNAs and plays a major role in the system's function. The pegRNA contains not only (i) the sequence complementary to the target sequence that directs nCas9 to its target site but also (ii) an additional sequence spelling the desired sequence changes (Fig. 1C).

In addition to the superior scope of possible edits, new generation prime editors (*e.g.*, PE3) have reduced indels at edit sites with a frequency of 0.86% compared with 2.5% for base editors. In terminally differentiated neurons *in vitro*, targeted insertions by prime editing were performed with an effective editing rate of 7.1%.^22^ Collectively, DNA base-editing and prime-editing tools could enable precise nucleotide substitutions in a programmable manner, without requiring a donor template.

DNA base editing and prime editing have remarkable potential as therapeutic tools to correct disease-causing mutations in the human genome. The ability to target nucleotides on either the plus or complementary minus DNA strand opens up the therapeutic applications of base editors considerably. More than 25% of human pathogenic single nucleotide polymorphisms (SNPs) can be corrected by targeting the four transition mutations, and in principle prime editing could correct up to 89% of pathogenic human genetic variants in ClinVar.^22^ Importantly, base editing employs cellular mismatch repair machinery and can be applied to reverse these defects in both dividing and terminally differentiated cells.

DNA base-editing may prove to be particularly well adapted for correction of mutations within large genes (>4 kb) where vector-mediated delivery is restricted by the cargo limits of AAV vectors.^5^ For instance, mutations in the *ABCA4* (coding sequence ∼6.8 kb) and *USH2A* (∼15.6 kb) genes together account for almost 25% of all inherited retinal diseases.^23^

Base editing can also be applied to autosomal dominant diseases, where gene supplementation is not a suitable method due to the requirement for silencing or ablation of the dominant-negative mutant allele. For example, base editing may present an attractive approach for targeted correction of the common P23H mutation in the rhodopsin gene (*RHO*) associated with adRP. Similarly, base editing may offer a novel therapeutic avenue in correction of heterozygous C > T mutations in *GUCY2D*, a leading cause of the autosomal dominant cone-rod dystrophy (CORD6). Bi-allelic knockout of the *GUCY2D* gene in adult mouse and NHP retinae showed thinning of the outer nuclear layer and external limiting membrane, which was not seen in the sham-treated eye.^24^ This has been attributed to excessive loss of retGC protein in the adult retina; thus, base editing could potentially overcome this issue by targeted correction of the mutant allele.

In addition, CRISPR-mediated base editing may provide a novel approach toward targeting the alternative complement pathway, which causes inflammation of the RPE. Recently, a base editing strategy to correct a common SNP (rs1061170) associated with age-related macular degeneration (AMD) risk in the complement factor H (CFH) gene showed 21.5% C-to-T nucleotide correction efficiency with no detected off-target effects in a HEK293A cell line expressing the pathogenic CFH variant.^25^ The therapeutic promise of prime editors may be especially useful in addressing the heterogeneity of IRD mutations. One foreseeable application is its use in *ABCA4*, which has a mutationally diverse but clustered mutation spectrum.^26^ Excision and replacement of mutation clusters would allow the correction of several, different mutations with a single approach.

Significant challenges remain with the delivery of therapeutic base editing and prime editing systems into retinal cells. A cytosine or adenine base editor plus a guide RNA totals ∼6 kb, whereas prime editors are >7 kb, both well beyond the packaging constraints of a single AAV vector. Using smaller Cas9-orthologs may help mitigate this, though delivery remains a challenge. Robustly assessing the method of delivery, long-term consequences of expression and editing efficiency *in vivo* will be imperative to advancing both base editing and prime editing to the clinic.

### Epigenetic editing

In addition to gene editing, CRISPR-Cas9 can be used for transcriptional regulation, in which catalytically inactivated “dead” Cas9 (dCas9) is fused to transcriptional effectors to repress genes directly (termed CRISPR interference or CRISPRi) or modified to act as a functional transcriptional activator (CRISPRa)^27^ (Fig. 1D). Multiple groups have shown that the mere binding of dCas9 to promoters and other regulatory regions can repress transcription by sterically hindering the RNA polymerase machinery.^27^ Nevertheless, the repressive capacity of the system is vastly improved when dCas9 is linked to a transcriptional repressor domain.^28^ CRISPRi can be used to knock down expression of pathogenic genes involved in retinal disease. The biggest advantage of CRISPRi is in its reversibility, as no permanent change is introduced into the genome. Thus, a transient delivery method, such as ribonucleoprotein (RNP) delivery, is capable of achieving a similarly transient effect.

Moreno *et al.*^29^ fused dCas9 to Krüppel-associated box (KRAB) for targeted repression of *Nrl*, a master regulator of rod photoreceptor determination. The authors demonstrated that *Nrl* knockdown could mediate reprogramming of rod cells into cone-like cells that are resistant to retinitis pigmentosa-specific mutations.^29^ Although this approach may reduce rod photoreceptor number and function and therefore lead to reduced night vision, relative preservation of the cones may allow patients to maintain visual acuity and central vision.

CRISPRi may have therapeutic potential in diseases caused by the development of choroidal neovascularization, such as wet AMD. For example, CRISPR-mediated epigenome editing may present a novel approach for robust and long-term suppression of vascular endothelial growth factor (VEGF-A) and reversal of the AMD-related phenotype (Table 1).^30^ Further, CRISPRi may reversibly silence the *RHO* (P23H) mutant allele in adRP,^17^ thus providing a safer alternative to indel-mediated mutant allele disruption, which might be associated with off-target disruption of the wild-type allele.

To date, applications of CRISPRa have been focused on gene screening but we can envision this approach as an alternative to gene supplementation. CRISPRa is useful for the treatment of haploinsufficiency, in which one copy of the gene is not sufficient to assure a normal phenotype, for example to overexpress the *PRPF31* gene in adRP.^31^ Although CRISPRa would provide an advantage in the case of genes that are too large to be delivered by AAV vectors, this approach potentially risks toxicity associated with gene overexpression. Despite their therapeutic promise, CRISPRa and CRISPRi may present additional limitations. Importantly, (i) CRISPRi lacks allele specificity since both alleles use the same promoter, (ii) both CRISPRa and CRIPSRi may have off-target effects on other genes with similar promoter sequences, and (iii) both require constitutive and long-term Cas9 expression for their therapeutic effects.

## Risks of Retinal Gene Editing

Genome editing using CRISPR-Cas9 systems *in vivo* carries a number of potential risks, in particular deleterious effects of off-target mutations, oncogenicity arising from the creation of DSBs, and immunogenicity associated with the gene-editing components. Therefore, the clinical viability of genome-editing therapies needs to be assessed on a risk–benefit basis.

### Potential off-target effects

The specificity of CRISPR-Cas systems is dependent on the complementarity of the 17–20 nt sgRNA as well as the fidelity of the endonuclease itself. Experimentally, off-target sites have been identified in human cell lines, with up to five or six mismatches to the guide RNA.^32^ A number of computer algorithms are available for predicting off-target mutation sites for CRISPR-Cas systems *in silico*. These tools are useful for sgRNA design but have limited accuracy, although this can be expected to improve in the future by using accumulated large datasets and machine-learning approaches.^33^

Experimental assays for unbiased detection of off-target mutations include *in vitro* genome-wide methods and cell-based methods.^34^ In addition, whole-exome sequencing or whole-genome sequencing may be used to assess the safety of retinal gene editing in preclinical studies involving NHPs with genomic DNA from the fellow eye as the reference sequence. Although each method has its own limitations, in practice, combinations of methods will be used at different stages of development to assess potential off-target effects of a genome-editing therapy.

Despite concerns over off-target mutations caused by CRISPR *in vivo*, preclinical studies by Maeder *et al.*^10^ showed that 84–88% of predicted off-target mutations were below the 0.1% detection threshold after SaCas9-mediated gene editing in human cell lines (U2OS and ARPE-19) and human retinal explants by using a combination of GUIDE-seq and Digenome-seq.^10^ This has been encouraging to the therapeutic genome-editing field, although long-term accumulation of even rare mutations remains a source of clinical concern. Cas9 mutagenesis and engineering is underway to generate endonucleases with greater on-target specificity and lower off-target effects, which could greatly assist in progressing CRISPR therapeutics.^15^

The expression of cytidine or adenine deaminases as part of DNA base editing constructs introduces additional sources of off-target effects. For instance, whole-genome sequencing of human-induced pluripotent stem cells stably expressing a cytidine base editor, AncBE4Max Apobec1 variant, demonstrated C-to-T and C-to-G mutations outside the *in silico* predicted off-target sites, which are compatible with the APOBEC mutagenesis signature.^35^ Moreover, cytidine and adenine base editors can target both DNA and RNA; thus, they are capable of causing transcriptome-wide off-target RNA editing off-targets, including self-editing of their own transcripts.^36^ This issue could be overcome by using engineered deaminases with reduced off-target RNA editing activity but retained on-target DNA editing activity.^36,37^

Compared with base editing, prime editing appears to be associated with lower levels of off-target mutations in human cell lines.^22^ This superior specificity may be the result of additional hybridization events required during prime editing: that is, hybridization between the reverse transcription template and the target DNA sequence. However, prime editing could potentially introduce small insertions at the target site, as the reverse transcriptase activity may extend a short way into the pegRNA scaffold beyond the primer sequence. In addition, caution surrounds the introduction of exogenous reverse transcriptase due to potential enabling effects on dormant pro-viral or retro-elements. Although early transcriptomic analysis of prime-edited human cell lines did not reveal any significant toxic effects, the clinical viability and safety of *in vivo* prime editing remain to be tested.^22^

### Concerns related to oncogenicity

A second consideration is the oncogenic potential of long-term Cas9 expression *in vivo*. Aside from rare but possibly deleterious effects of off-target mutations on tumour suppressor genes, Cas9-induced DSBs could potentiate chromosomal translocation or chromosomal integration of the AAV genome.^5^ In addition, Cas9-induced DSBs trigger cell cycle arrest in immortalized human RPE cells via the activation of the tumor suppressor, p53.^38,39^ Although this could potentially drive positive selection of p53-deficient cells within a dividing population (*e.g.*, during *ex-vivo* editing of hematopoietic stem cells), it seems unlikely to be of significant concern in retinal genome editing of terminally differentiated photoreceptors and usually non-dividing RPE. On the contrary, transient inhibition of p53 has been shown to be a potential adjunct for increasing the rate of homologous recombination from a donor template over NHEJ; thus, it may be beneficial in prime editing.^38^

### Immune and inflammatory responses

The origin of CRISPR-Cas9 components in common bacteria raises the possibility of intraocular immune and inflammatory responses to *in vivo* retinal gene editing, which could lead to cell damage and reduce clinical efficacy. Pre-existing antibodies to SaCas9 and SpCas9 were found in 78% and 58% of humans,^40,41^ as well as NHPs.^10^ However, no significant inflammation has been observed in NHPs after subretinal administration of AAV5 vectors encoding a CRISPR-SaCas9 gene-editing construct targeting *GUCY2D* in autosomal dominant CORD.^24^

Increases in AAV-neutralizing antibodies and Cas9-specific T-cell responses correlated with the number of subretinal injection blebs created. Neither the pre-existing or induced antibody and T cell responses resulted in significant intraocular inflammation in the treated animals, nor did they limit the level of gene editing. In fact, the animal with the highest pre-dose T cell response to Cas9 showed the highest editing efficiency in the *GUCY2D* gene-editing study.^24^ Similarly, subretinal delivery of AAV5-encoded CRISPR-SaCas9 to correct the common deep-intronic mutation in *CEP290* associated with LCA10, called EDIT-101 (Editas Medicine, Inc.), induced only mild immune responses in NHPs without the need for systemic immunosuppression.^10^ The inflammatory responses correlated with levels of antibodies to AAV5, but once again the NHPs with the highest level of pre-existing anti-SaCas9 T-cells showed the greatest editing efficiency.

## Strategies to Control Cas9 Activity and Improve Safety

**Figure 2.**
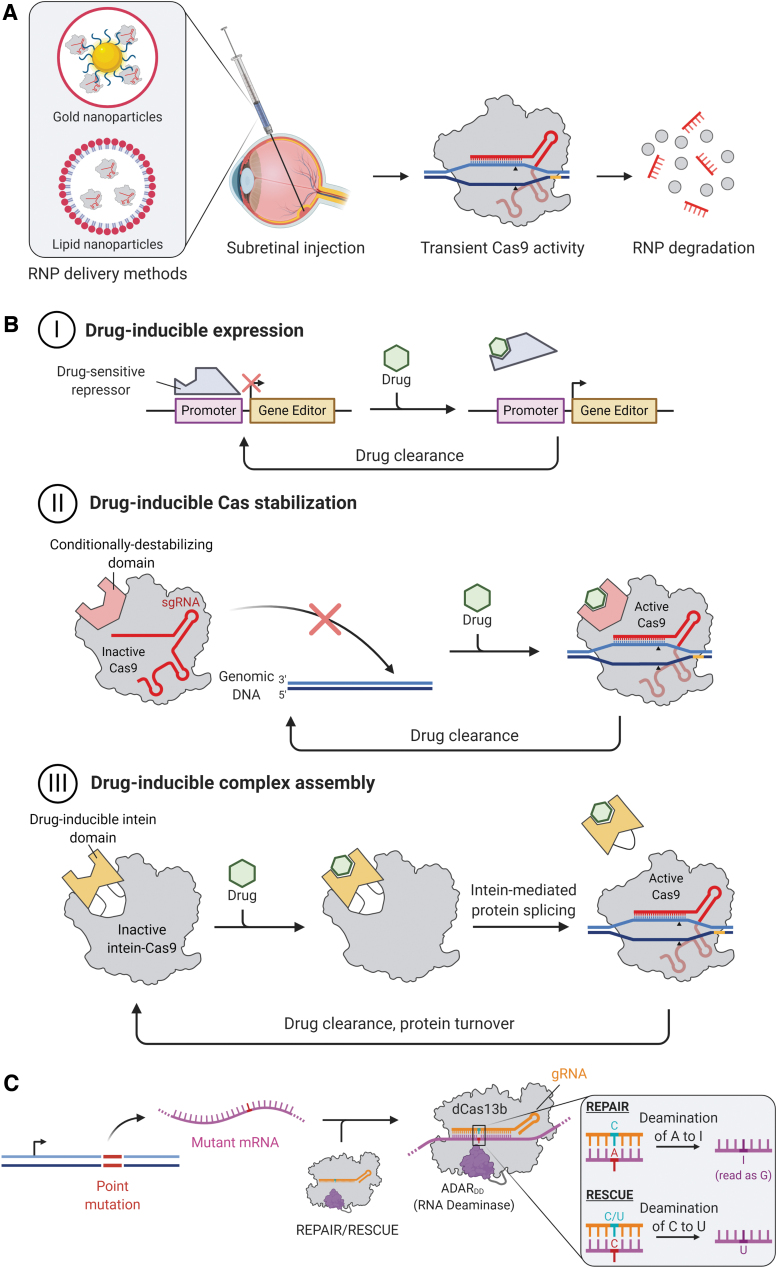
Strategies to mitigate the risks of off-target editing. **(A)** Delivery of Cas9-sgRNA RNPs. RNPs can be packaged into synthetic gold or lipid nanoparticles for delivery by injection to the subretinal space. Nanoparticles that are taken up by retinal cells release the RNPs to perform gene editing until they are naturally degraded, providing transient activity to minimise off-target editing. **(B)** Temporal control of gene editing with small-molecule drugs. Narrowing the time window for gene editor activity can significantly reduce off-target editing and may be achieved with small-molecule-inducible systems. Successfully implemented systems include: **(I)** inducible expression of editing components through drug-sensitive repressors; **(II)** inducible stabilization of Cas9 by insertion of a ligand-binding domain into Cas9; and **(III)** inducible complex assembly by insertion of a drug-responsive intein domain. **(C)** RNA editing with REPAIR/RESCUE. A catalytically dCas13b is fused to the RNA deaminase domain from ADAR (ADAR_DD_). The fusion utilizes a gRNA to locate the target RNA sequence and allow the deaminase to convert a pathogenic point mutation (shown in *red*) from A to I or C to U in the case of REPAIR and RESCUE, respectively. Incorporating a mismatched C or U into the desired position of the guide sequence (shown in *light blue*) improves the specificity of editing. dCas13b, dead Cas13b; RNP, ribonucleoprotein. Color images are available online.

As most of the safety concerns of therapeutic genome editing relate to persistent Cas9 activity *in vivo*, a variety of strategies may be developed to facilitate transient or controlled Cas9 expression (Fig. 2).

### Delivery of Cas9-sgRNA RNPs

Cas9-sgRNA RNPs degrade after about 3 days in RPE cells.^30^ To achieve transient genome-editing activity, non-viral vectors should be considered as they offer low risks of immune reactions or insertional mutagenesis and have a large delivery capacity for bulky RNPs, DNA, or RNA constructs (Fig. 2A). Indeed, non-viral strategies based on physical methods (including electroporation, gene gun, nucleofection) and chemical methods (including nanoparticulated systems) have achieved considerable progress. Further, conjugation of RNPs or nucleic acids to cell-penetrating peptides may further improve delivery of large molecules into retinal cells.^42^

To date, however, the majority of non-viral delivery methods are not applicable for clinical gene delivery due to low efficiency or toxicity. The use of such methods to deliver plasmid sequences containing large transgene elements also raises safety concerns over uncontrolled dissemination of antibiotic-resistant genes or other bacteria-derived sequences. Minicircles are plasmid derivatives that are devoid of bacterial backbone elements and are known to increase transfection efficiencies likely due to their smaller size and, thus, are more adept to achieve therapeutic gene expression.^43^ Minicircles have proven to be a reliable tool for efficient transgene expression in eukaryotic cells both *in vitro* and *in vivo*, as well as for *ex vivo* modification in a range of cell and gene therapy applications, including photoreceptors.^44^ Their potential application to retinal gene editing remains to be assessed.

### Drug-inducible Cas9 activity

In terms of viral vector-mediated genome editing, inducible Cas9 constructs could enable transient and regulated Cas9 expression, thus preventing long-term accumulation of off-target mutations (Fig. 2B). Temporal control of Cas9 expression may be achieved by using transient or drug-inducible promoters, for example, the steroid-inducible *GRE5* promoters.^45^ Clinical viability of steroid-inducible promoters is facilitated by the availability of ready-approved corticosteroid eye drops, periocular injections, and intraocular implants, which would also provide local immunosuppression after retinal gene therapy. However, concerns over leaky expression with steroid-inducible promoters must be considered, as even very low basal expression of Cas9 may cause accumulation of off-target mutations over time.

One drug-inducible promoter that could provide tighter transcriptional control of Cas9 expression is the doxycycline (or tetracycline)-inducible Tet-ON system. Tet-ON-regulation of both Cas9 and sgRNA expression has afforded temporal control of gene editing in cells.^46^ These serve as valuable proof-of-concept for temporal control of gene editing, but *in vivo* delivery of an effective drug-inducible gene-editing system is yet to be described. This is likely due to the challenge of delivering the exogenous transcription regulatory components, such as the reverse tetracycline transactivator, in addition to the Cas9 and sgRNA. Despite the improvement in leaky expression that Tet-ON systems offer over steroid-inducible systems, however, they are not leak-free and may raise similar concerns over accumulation of mutations over time. Although lentiviral vectors could be used to deliver Tet-ON systems to certain cell and tissue types, they are typically poor transducers of photoreceptors and are unlikely to achieve sufficient efficacy for therapeutic retinal gene editing.^46^

An alternative strategy for temporal control of Cas9 activity is small-molecule induction at the post-translational level, with a number of such systems having been described.^47^ Drug-induced Cas9 protein degradation by ubiquitin-ligase recruitment has been suggested,^47^ however this would require continual drug delivery. In contrast, small-molecule-activated Cas9 proteins appear more applicable to clinical genome editing. One promising strategy is to insert a 4-hydroxytamoxifen (4-HT)-responsive intein domain within the Cas9 nuclease, which facilitates internal protein splicing to produce an active Cas9 only in the presence of tamoxifen.^48^ Alternatively, insertion of the estrogen receptor-α (hERα) ligand-binding domain within Cas9 to enable allosteric protein stabilization only in the presence of 4-HT has also been shown.^49^

The hERα ligand-binding domain has also been fused to the termini of Cas9, leading to its sequestration within the cytoplasm until 4-HT-activated nuclear translocation allows genome editing to occur.^50^ These systems have demonstrated the increased specificity achievable with inducible Cas9. However, they are currently associated with reduced on-target editing efficiency compared with the wildtype counterparts, likely due to alterations in Cas9 structure. Moreover, the systems have been based on the large SpCas9 fused to other bulky components, making them challenging to deliver by using AAV vectors. Although these approaches hold promise, future optimization efforts are required to achieve a deliverable and therapeutically efficacious drug-responsive Cas9 protein.

### Self-destructing CRISPR-Cas9

Self-destructing CRISPR systems incorporating a self-targeting sgRNA have been proposed and shown to be viable for retinal gene editing without compromising on-target efficacy.^51^ It remains to be seen whether such systems could provide complete inactivation of Cas9 activity *in vivo* and how they compare with transcriptional and post-translational regulatory systems.

### mRNA editing

Finally, editing mRNA rather than DNA may circumvent the risks associated with genome editing, as off-target events would not result in permanent mutations.^52^ The majority of RNA editors utilize the deaminase domain from the human adenosine deaminase acting on RNA type 2 (ADAR2_DD_), which converts adenosine to inosine predominantly in protein-coding regions of pre-mRNA in its native role. The newly formed inosine is then interpreted as guanosine when translated by the ribosomal machinery.^52^ Fusing ADAR2_DD_ to an RNA-guided RNA-binding domain facilitates site-specific A-to-I editing in mRNA.

Various RNA-guided ADAR2_DD_ systems have been explored, for example, the fusion of ADAR2_DD_ to a catalytically dead RNA-targeting Cas13b (dCas13b) (Fig. 2A). Cox *et al.* developed REPAIRv1 by fusing dCas13b from *Prevotella sp. P5-125* (dPspCas13b) to ADAR2_DD_(E488Q), a hyperactive mutant with increased A-to-I editing efficiency.^53^ An additional T375G mutation in the ADAR2_DD_ domain was found to increase specificity 919-fold with a modest decrease in on-target efficiency, yielding REPAIRv2. The ADAR2_DD_-dCas13b approach was recently extended to cytidine-to-uridine editing. Rational mutagenesis of ADAR2_DD_ to generate cytidine deaminase activity and subsequent fusion to dCas13b from *Riemerella anatipestifer* (dRanCas13b) yielded RESCUE (RNA Editing for Specific C-to-U Exchange).^54^ Together, REPAIR and RESCUE provide scope for correcting up to 61% of pathogenic point mutations within mRNAs.^52^ Further, C-terminal truncation of dPspCas13b and dRanCas13b in REPAIR and RESCUE, respectively, affords active RNA editors that are small enough to be packaged into single AAVs.

One of the key challenges for therapeutic RNA editing is the need for stable and long-term expression of the editing construct within the target cells. Evaluation of these and other mRNA editing approaches *in vivo* will be required to gauge their feasibility of delivery and clinical efficacy.

## Moving Retinal Gene Editing Toward Clinical Application

The proven safety and clinical success of AAV vector-mediated gene supplementation in treating recessive IRDs associated with loss-of-function mutations^3,6,7^ suggests that new gene-editing strategies would be best applied to mutations unamenable to this approach. Mutation-specific genome-editing approaches should, in the first instance, be expected to focus on targeting the most frequent IRD-associated mutations in genes that are beyond the coding capacity of single AAV vectors. In addition, gene-editing therapy is likely to be most useful when there is a need to correct or silence a dominant-negative or gain-of-function mutation. Such dominant-negative or splicing mutations may also be amenable to treatment using intravitreally delivered ASO therapies, which should be considered as a potentially safer alternative to permanent genome editing.^9^

For mutations that cause haploinsufficiency, base editing, prime editing, or CRISPRa may be considered, although technical challenges associated with delivering these systems into retinal cells remain. More generic approaches for slowing disease progression or restoring visual function in IRDs are also under exploration, including AAV-mediated neuroprotection^55^ and reprogramming of photoreceptors through AAV-CRISPR/Cas9-mediated knockdown of *NRL*.^56^ Moreover, targeting common neurodegenerative and neuroinflammatory disease pathways involved in the progression of IRDs, age-related macular degeneration, and glaucoma need to be explored.

The CRISPR/Cas systems that are capable of targeted knockdown of disease alleles and introducing desired genomic alterations have created a renaissance in the gene therapy field. The advent and rapid development of base editing, prime editing, and mRNA editing are expected to make gene editing safer in the future. With only limited changes to the guide RNA sequence while keeping the rest of the gene-editing system identical, a large number of pathogenic mutations associated with inherited retinal disease may be corrected. This makes gene-editing technologies attractive for the clinical development pathway, especially given the heterogeneous nature of IRDs.

**Figure 3.**
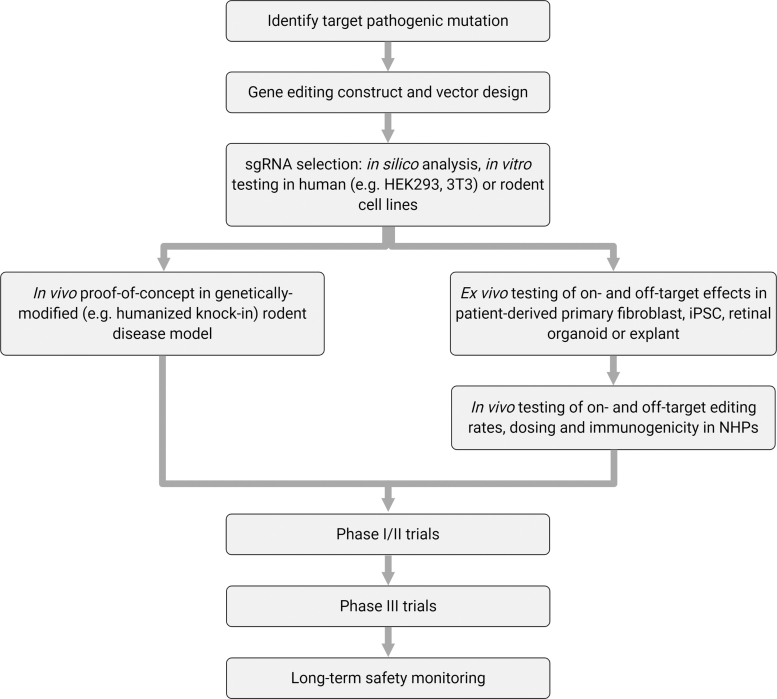
Pathway for developing retinal gene-editing therapies.

One of the key considerations in testing gene-editing therapies is that the primate-specific nature of therapeutic sgRNAs requires preclinical testing to be conducted in human tissue and NHPs; thus, the costs involved in the development of such therapies will be substantial (Fig. 3). To this end, developments in human retinal organoids will likely prove useful in simplifying and reducing costs of preclinical evaluation of retinal gene-editing therapeutics. In addition, once a few prototypic retinal gene-editing systems have undergone rigorous *in vivo* testing in NHPs or humans,^10,24^ future clinical development pathways may become more simplified.

## Conclusions

The AAV vector-mediated gene supplementation has demonstrated the feasibility of therapeutic gene therapy in the retina, whereas CRISPR/Cas9 systems can potentially allow a much broader range of inherited retinal diseases to be treated. Although the preclinical studies of EDIT-101 have delivered highly encouraging results, much attention will now be focused on the outcomes of the phase I/II clinical trial, which has dosed its first patient in early 2020. *In vivo* gene editing poses new challenges associated with drug delivery and questions with regards to long-term safety. Currently, the field is undergoing rapid development with a number of competing gene-editing strategies, including allele-specific knockdown, base editing, prime editing, and RNA editing, that are under investigation, with each offering a differing balance of on-target editing efficiency versus off-target risks. Moreover, a variety of strategies to improve temporal control of gene-editing systems *in vivo* need to be explored to minimize long-term risks. Testing these newly developed CRISPR technologies in human retinal tissue, organoids and *in vivo* will help to highlight the most viable therapeutic approaches for treating inherited retinal diseases in the future.
